# An Interesting Case

**Published:** 1895-11

**Authors:** D. W. Gilbert

**Affiliations:** Katonah, N. Y.


### AN INTERESTING CASE.

By D. W. GILBERT, V.S.,
KATONAH, N. Y.

PATIENT, a bay gelding, which the owner stated was unable to eat, he thought from a defective tooth. A copious flow of saliva dribbled constantly from the mouth.

An examination, which was somewhat difficult owing to great uneasiness of the animal, revealed a foreign body firmly lodged between the fourth and fifth superior molars—a piece of spruce board (including a knot about three and one-half inches long), probably having been there for several days, as it had become of very offensive odor. The cause of this unique traumatism was explained by the fact that the animal had recently accomplished the feat of gnawing away nearly all the front portion of his stall. Recovery was prompt and complete.

